# Population Landscape of Familial Cancer

**DOI:** 10.1038/srep12891

**Published:** 2015-08-10

**Authors:** C. Frank, M. Fallah, J. Sundquist, A. Hemminki, K. Hemminki

**Affiliations:** 1 Division of Molecular Genetic Epidemiology, German Cancer Research Center (DKFZ), Im Neuenheimer Feld 580, D-69120, Heidelberg, Germany.; 2 Center for Primary Health Care Research, Lund University, 205 02 Malmö, Sweden.; 3 Stanford Prevention Research Center, Stanford University School of Medicine, Stanford, California 94305-5705, USA.; 4 Cancer Gene Therapy Group, Department of Pathology and Transplantation Laboratory and Haartman Institute, University of Helsinki, Finland.

## Abstract

Public perception and anxiety of familial cancer have increased demands for clinical counseling, which may be well equipped for gene testing but less prepared for counseling of the large domain of familial cancer with unknown genetic background. The aim of the present study was to highlight the full scope of familial cancer and the variable levels of risk that need to be considered. Data on the 25 most common cancers were obtained from the Swedish Family Cancer Database and a Poisson regression model was applied to estimate relative risks (RR) distinguishing between family histories of single or multiple affected first-degree relatives and their diagnostic ages. For all cancers, individual risks were significantly increased if a parent or a sibling had a concordant cancer. While the RRs were around 2.00 for most cancers, risks were up to 10-fold increased for some cancers. Familial risks were even higher when multiple relatives were affected. Although familial risks were highest at ages below 60 years, most familial cases were diagnosed at older ages. The results emphasized the value of a detailed family history as a readily available tool for individualized counseling and its preventive potential for a large domain of non-syndromatic familial cancers.

Hereditary cancer has become an important issue in oncology clinics because of the success in implementing genetic testing and/or screening methods for cancer syndromes. Most importantly, this information has been useful in preventing new tumors and cancer deaths^1^,^2^. However, hereditary cancer syndromes with identified high-risk genes account only for a small proportion of familial cancers while familial aggregation has been suspected for practically all cancers many of which lack a fully dissected genetic background^3^. Public awareness of familial cancer has increased and the demand for counseling has been a challenge to the oncology community, firstly, on how to obtain and judge the family history of various cancers and, secondly, how to estimate the risk and propose management. In the general public the topic of familial cancer risk is characterized by apprehensiveness which may explain why poorly understood familial predisposition is overlooked or ambiguously communicated in the recommendations of many professional organizations. The American Cancer Society considers a family history an indication for screening or surveillance only for cancers of the breast, prostate, colorectum, endometrium, and ovary but the focus is on mutation testing^4^. Similarly, the recent American Society of Clinical Oncology expert statement was strictly limited to cancer syndromes with known gene defects and the much larger domain of familial cancer with poorly defined genetics was not even mentioned^5^. Such a demarcation leaves 90% of familial cancers unattended, including prostate, lung and bladder cancers, and non-Hodgkin lymphoma for which gene testing may not be available^6^. With the recent emphasis on gene testing and the visions offered by next generation sequencing^7^, there is a danger that the valuable and readily available anamnestic information on family history remains unused, as if the concept of ‘individualized medicine’ would be restricted to the genetic make-up. A negative gene test does not overrule familial risk when no deleterious mutations have been found in the family. Therefore, there is no substitute for medical caregivers taking meticulous family histories. While generally there is little well documented information of this issue, the available studies suggest that less than half of medical charts in the USA had any family history; even if recorded, the data were superficial, e.g., only dichotomous (yes or no) and lacking diagnostic ages^8^,^9^. In Europe, the situation is probably no better and in continental Europe counseling tends to be limited to gene testing. A contributing problem is that empirical data on familial risks have been limited; i.e., physicians have not been aware of the relevance of familial cancer risk for the patient being attended to, and thus obtaining the anamnesis has not been of sufficiently high priority to be included in medical records^10^.

In the present article we aimed to describe a nationwide landscape of familial cancer by defining the risk by the number and diagnostic age of affected family members, each factors that need to be considered in clinical genetic risk assessment. The analyses are based on the latest version of Swedish Family-Cancer Database (FCD 2010) which is a unique source for family studies comprising almost 15 million people with clinical cancer data and other detailed personal information from 1958 through 2010.

## Methods

### Data source and patients

The FCD was used to estimate familial cancer risks for the 25 most common cancer sites. The database comprises information from the Multigeneration Register, censuses and death notifications provided by Statistics Sweden, and information from the Swedish Cancer Registry. In its latest update from 2010 the database included 14.7 million individuals, where all people born in Sweden from 1932 onwards (the offspring generation) were registered with linkage to their biological parents (the parental generation).

More than 1.7 million medically verified cancer cases were recorded in the Cancer Registry from 1958 to 2010. The 7^th^ revision of the International Classification of Diseases (ICD-7) was used to identify the cancer type. Death information is assumed to be complete from 1961 onwards. In order to investigate cancer risks for offspring of affected parents as well as risk shared by siblings, all individuals with identified parents and non-missing information on birthday and sex were selected for the analysis, totaling in 8,148,737 index individuals. Among them 343,494 were diagnosed with one of the cancer sites under study.

### Relative risk estimation

Familial risk was assessed for the offspring generation by estimating relative risks (RRs) in terms of incidence rate ratios using a Poisson regression model^11^. Hereby, incidence rates for people with a concordant cancer in a first-degree relative (FDR, positive family history) were compared to the corresponding rates for those individuals who had no concordant cancer among their FDRs (negative family history). Cases and person-years were counted first according to family history and stratified for sex, age group, calendar period, residential area, and socioeconomic status to account for potential confounders. These variables were used as covariates for the model building whereby cases were assumed to follow a Poisson distribution and person-years were included as offset^12^. The ratio of the fitted mean given a positive family history divided by the corresponding estimate for negative family history yielded the RR. Wald estimates were available to test for the significance of family history as a risk factor and to provide confidence intervals (CIs) for the RR. These were scaled using the Pearson chi-square estimate to account for potential dispersion^13^. Familial risk was considered to be significantly increased or decreased, respectively, if 1.00 was not included in the RR’s 95% CI which is equivalent with testing the significance of the regression coefficient for family history at the 5% confidence level.

### Different levels of family history

In particular, independent groups for certain familial relationships were considered in different settings in order to examine the differences in the familial risk shared by parents and siblings, the dependence on diagnostic age, and the risk shared by multiple affected relatives. Risks for parental probands were calculated twice: First, with no limit on the parental diagnosis age and second, regarding only parental cancers if they were diagnosed until the age of 78 years. This cut-off was used for comparison reasons since it equaled with the maximum age for siblings in the FCD.

The dependence of familial risk on diagnostic age was investigated considering FDRs in general. We distinguished between cancer diagnosis before and after the 60th birthday for both index persons and their relatives. The corresponding diagnostic age specific RRs were tested for equality by a likelihood ratio test. Familial risks were also analyzed to check for differences when both parents, one parent and one sibling, or no parent but multiple siblings were affected. This analysis was limited to a subgroup of most common cancer sites due to sample size constraints.

### Follow-up

The follow-up period started from the beginning of 1961, the birth year, or the immigration year, whichever came latest. The follow-up was terminated when a person was diagnosed with cancer, emigrated or died, or at the end of 2010, whichever came first. The register-based definition of period at risk was used for person-year calculations whereby a person was considered to be at familial risk irrespective of when family members were diagnosed with cancer^14^.

## Results

### Risk estimates for siblings and offspring of affected patients

Familial risks were analyzed for 25 different cancer sites for which at least 10 affected parent-offspring and sibling pairs were detected. All cancer types under study showed significantly increased risks at 5% confidence level for offspring when their parents or siblings were affected, as shown in Table 1. Parents were used as probands with and without the age limit of 78 years (i.e., offspring maximal age), and for most cancers the RRs were only slightly higher if the parental age was limited. While the RRs for offspring of affected parents ranged between 1.5 and 2.5 for the majority of cancers, those for testicular cancer (3.90 and 3.96 with age limit, respectively), small intestine cancer (4.81–5.34), and cancer of the thyroid gland (5.13–5.61) were markedly higher. RRs for siblings were considerably increased not only for the above sites (testicular cancer 6.94, small intestine cancer 10.11, thyroid gland 5.43), but also for Hodgkin lymphoma (9.60). Sibling risks were higher than risks in offspring of affected parents (irrespective of parental age) for all cancer sites, except for endometrial cancer and cancers of the thyroid and other endocrine glands. Nonetheless, significance differences at the 5% level were only detected for stomach, colorectal, lung, prostate and testicular cancers, and Hodgkin lymphoma.

### Early and late onset of familial cancers

Analyzing the data for familial risk for cancers diagnosed at early and late ages (Table 2), the results revealed increased familial risks for almost all cancers regardless of whether individual or relative diagnostic age was below or above 60 years. Even for familial risks which did not appear significantly increased (p-value ≥ 0.05), the RRs were greater than 1.00, except for elderly individuals with cervical cancer. The risk was highest for almost all cancers if index individuals and their relatives were affected at an early age. For some cancers the RRs seemed to decline in the order: young index persons/young relatives, old index persons/young relatives, young index persons/old relatives, and both old index persons and relatives. Taking an example for prostate cancer, the RR declined in order 7.21, 3.51, 2.79, and 2.13. However, the respective familial cases increased in order 673, 916, 2727, and 7300. A trend test cannot be applied for these kinds of data but we tested the equality for RRs in the four independent age groups by a likelihood ratio test. In fact, RRs differed significantly from each other among age groups for the majority of cancer types.

It is noteworthy that among 23 cancer types with cases in the highest age category 19 showed a significant increase. Among the four remaining cancer types the sample size for cervical and thyroid cancer was small and the RR for kidney cancer was almost significant (RR = 1.29 with corresponding 95% CI 0.98–1.71). In agreement with prostate cancer, the other most common cancers, including colorectal, lung, bladder, and skin cancers showed the largest number of affected individuals in the highest age group and each with a significant familial risk. The exception for case numbers was breast cancer but the familial risk remained significant in the highest age group.

### Familial cancer risks in multiplex families

Familial cancer risks when two or more FDRs were affected (i.e., multiplex families) with a concordant cancer and corresponding test statistics for significant differences among RRs were presented in Table 3. RRs for breast cancer (9.49) and melanoma (6.00) were strikingly increased when both parents were affected and the estimates differed significantly from those calculated for one affected parent in Table 1. If one parent and one sibling were affected, risks for cancers of the prostate (4.59) and the nervous system (9.47) and of melanoma (8.20) were two to five times higher compared to RRs for one affected FDR (Table 1). For all but lung and bladder cancers, the risks for individuals with an affected parent and a sibling were significantly higher than the risks for cases with one affected parent or sibling (all p-values < 0.0001). The risk estimates did not differ significantly between patients with one affected parent and sibling and patients with two affected siblings. The risk for prostate cancer (5.25) and melanoma (4.47), when two siblings were affected, was about twice as high as the risk when one sibling was affected but the difference was only significant for prostate cancer. In the case of three affected siblings, the case numbers did not allow for reliable risk estimates, except for prostate cancer featuring an exceedingly high RR of 6.78.

### Proportion of familial cancers among all cancers

The highest proportions of familial patients diagnosed at the age of 60 years or older were found for cancers of the prostate (21.9%), breast (14.4%), colorectum (13.8%), and lung (10.3%) as presented in Table 4. Apart from these types, almost all remaining cancers showed percentages less than 5%. An overwhelming proportion (>90%) of familial cancers were in families of two patients, with the exception of prostate cancer (87%). Families with three or more concordant cases accounted for more than 1% of all cancer only for prostate (2.9%) and breast cancer (1.1%).

## Discussion

Our study showed that the existence of affected family members is an important risk factor for all cancer types. For common cancers familial risk even increased with the number of affected family members. Until now, over 100 cancer predisposing variants have been identified and in addition over 300 low-risk loci have been mapped^15^. However, only a small proportion of familial cancer can be explained by the established genetic predisposition, and the proposed risk estimates vary extensively^16^. For example, in colorectal cancer some studies have assumed that mismatch repair gene defects (hereditary non-polyposis colon cancer) account for most of familial aggregation^17^, but the recent exome sequencing data put the figure at 11% of familial cancer and early onset cases^18^. In clinical genetic counseling mutation testing is offered only for a few high-risk cancer predisposing genes. In Europe, the UK, Netherlands, and the Nordic countries offer genetic counseling services to some degree on cancers for which predisposing genes are not known but in the main continental Europe such counseling is rare. In Germany, guidelines for testing genetic predispositions emphasize on breast-ovarian and colorectal cancers^19^.

Familial risk was assessed based on the FCD which is the world’s largest database for familial studies allowing for unbiased risk estimates in terms of selection and recall bias as the whole Swedish population was covered with registered data on family relationships and medically verified data was available^20^. We showed that the cancer risks conferred by affected parents or siblings were about 2-fold compared to the risk for individuals with unaffected relatives. If a parent was affected, risks for offspring were highest, some 5-fold for cancers of the thyroid gland and small intestine. If a brother or sister was affected with small intestinal cancer or Hodgkin lymphoma the risk was increased 10-fold. Our previous study has shown a low correlation of familial risks in common cancers between spouses, suggesting that shared environment in adulthood is not an important risk factor for familial risk, with the exception of tobacco-related cancers such as lung cancer^6^. In this study, slightly higher risks for concordant cancers among siblings than for offspring of affected parents may indicate recessive genetic effects or deleterious influence of shared environmental risk factors during childhood and adolescence^21^. For some cancers such as stomach, lung and prostate cancers, and Hodgkin lymphoma these differences were highly significant. Stomach cancer with sharp decline in incidence would show much higher rates in the parental than in the offspring generation^22^. For smoking, childhood environmental exposure is likely to facilitate persistent addiction^23^. For prostate cancer, in addition to the possible recessive effects, diagnosis of cancer in a brother may alert other brothers to seek medical contacts while diagnosis in a father may be of less concern^24^. For Hodgkin lymphoma, shared childhood socioeconomic environment has been offered as an explanation^25^.

We observed that familial RRs depend on age for most but not all cancers. This effect was already detected for some common cancers in a study based on a previous version of the FCD^26^. Now, we had a sufficiently large sample size to investigate even rarer cancers and most of them showed significantly different risks, as tested between the age groups, depending on the individual age and the family member’s diagnostic age. For almost all cancers the individual cancer risk was highest below 60 years of age if the affected FDR was also diagnosed at that age. The risk declined considerably for elderly individuals if relatives were also diagnosed at late ages which was most obvious for colorectal and prostate cancers. For the latter, the corresponding RRs were reduced from 7.21 to 2.13 from early to late diagnostic age. Interestingly, this seemingly trend appeared to be reversed for myeloma, yet with small case numbers.

A high familial risk at an early age, shown above for most cancers, is in accord with the current understanding of cancer syndromes which disproportionately afflict young individuals^27^. However, the seemingly opposite information that significant familial risks and most familial cases in many cancers are in fact found in individuals older than 59 years (when family members were also diagnosed at high age) has implications with regard to clinical counseling: counselor should not overlook cancers diagnosed at an advanced age. A contributing reason for decreasing familial risks in the elderly population is the high background incidence^28^.

Familial relative risks were further increased if multiple FDRs were affected. For melanoma, risk shared by parents was almost identical to sibling risks, but it was more than doubled if both parents or a parent and a sibling were affected. The aggregation of affected family members in these few families may be due to genetic predisposition through the high-risk susceptibility gene CDKN2 or, to lesser extent, by shared modifiable risk factors such as UV radiation, as melanoma is known to be a heterogeneous disease with differing etiologies^29^. The risk for nervous system tumors was more than 5-fold higher if a parent and a sibling were affected compared to the risk conferred by affected parents or siblings only. This remarkable increase was already observed elsewhere^30^. Since ionizing radiation is the only thus far established environmental risk factor for nervous system cancer^31^, the majority of familial cases where two family members were affected is probably caused by low-penetrance genes while rare cancer syndromes such as Li-Fraumeni, neurofibromatosis 1 and 2, von Hippel–Lindau, tuberous sclerosis, Turcot, and Gorlin would account for multiplex families^32^,^33^. The proportional increase in prostate cancer risk and number of affected relatives support the hypothesis of rare autosomal dominant susceptibility genes^34^. Furthermore, high-risk cancer predisposing genes such as BRCA1/BRCA2 associated with familial breast cancer or mismatch repair genes involved in Lynch syndrome (hereditary non-polyposis colon cancer) may account for significantly increased risk of breast and colorectal cancers in the cluster of affected parents and siblings. Interestingly, the corresponding risks for two affected siblings were also elevated, but only modestly and the difference was significant only for breast cancer. Successful screening of family members of mutation carriers may have prevented some breast and colorectal cancers in recent decades^1^,^2^. In families where both parents and offspring suffer from breast cancer, mutations in the *BRCA1* gene, which were estimated to account for 60% to 76% of male breast cancers in high-risk families, are most likely to be causative^35^.

Most familial cancers were diagnosed at the age of 60 years or older. Among them, the number of families where 3 or more family members were diagnosed with a concordant cancer was negligible, with the exception of the most common cancers of the prostate and breast. The low proportion of families with 3 or more affect individuals is likely to define the genetic architecture of familial cancer; high-penetrance predisposition is rare compared to low-penetrance risk disposition signaled by two-case families.

In conclusion, our results show that familial risk is a shared feature of all cancers and for many cancers multiple affected family members signal a high or very high risk that would necessarily require medical action. Some of such families are likely carriers of known high-risk cancer predisposition genes. However, the major proportion of familial cluster is probably caused by genes that remain to be discovered. Nevertheless, medical or behavioral intervention may be indicated, including screening recommendations or avoidance of carcinogenic exposures. The readily available information of family history deserves more attention in the first oncology contacts and established referral mechanisms for clinical counseling to evaluate screening and prevention strategies individually tailored to patients and their family members.

## Additional Information

**How to cite this article**: Frank, C. *et al*. Population Landscape of Familial Cancer. *Sci. Rep.* **5**, 12891; doi: 10.1038/srep12891 (2015).

## Figures and Tables

**Table 1:** Familial cancer risks conferred by affected parents and siblings.

Cancer site	Patients with negative family history	1 parent affected			1 parent (age limited) affected^1^			1 sibling affected			p-value for difference between RR_sib_ and RR_Par_^2^
		N^3^	RR^4^	95% CI^5^	N	RR	95% CI	N	RR	95% CI	
Upper aerodigestive tract	7236	136	1.51	(1.13–2.02)	113	1.57	(1.14–2.15)	56	1.73	(1.10–2.72)	0.72
Esophagus	2248	26	2.45	(1.55–3.90)	18	2.33	(1.34–4.05)	12	3.36	(1.70–6.62)	0.41
Stomach	4411	222	1.72	(1.47–2.02)	172	1.78	(1.49–2.13)	46	2.97	(2.11–4.18)	0.0090
Small intestine	1489	17	4.81	(2.99–7.75)	14	5.34	(3.16–9.01)	14	10.11	(5.98–17.09)	0.09
Colorectum	25187	2945	1.72	(1.64–1.80)	2100	1.80	(1.71–1.90)	905	2.00	(1.84–2.17)	0.0294
Liver	4625	130	1.61	(1.29–1.99)	101	1.78	(1.39–2.27)	30	2.07	(1.32–3.23)	0.56
Pancreas	5152	178	1.96	(1.66–2.33)	140	2.09	(1.73–2.54)	54	2.73	(2.01–3.71)	0.15
Lung	18111	1346	1.86	(1.74–1.99)	1108	1.92	(1.78–2.07)	712	2.50	(2.28–2.74)	**<.0001**
Breast	56566	6102	1.73	(1.67–1.80)	4989	1.78	(1.70–1.85)	3493	1.84	(1.75–1.94)	0.24
Cervix	8074	128	1.57	(1.28–1.93)	122	1.59	(1.29–1.96)	54	2.03	(1.48–2.78)	0.21
Endometrium	9298	301	2.21	(1.88–2.61)	257	2.37	(1.98–2.83)	128	2.13	(1.66–2.73)	0.49
Ovary	8633	261	2.52	(2.20–2.88)	241	2.67	(2.32–3.07)	122	2.97	(2.44–3.61)	0.38
Prostate	37805	6622	2.10	(2.03–2.18)	4469	2.33	(2.24–2.43)	3390	2.59	(2.48–2.72)	**0.0005**
Testis	6652	32	3.90	(2.63–5.79)	32	3.96	(2.67–5.86)	82	6.94	(5.42–8.89)	**0.0173**
Kidney	7739	201	1.58	(1.34–1.87)	175	1.68	(1.40–2.01)	75	2.09	(1.59–2.75)	0.18
Urinary bladder	10944	513	1.76	(1.56–1.98)	370	1.84	(1.61–2.12)	172	1.90	(1.55–2.32)	0.81
Melanoma	22807	763	2.43	(2.23–2.66)	643	2.55	(2.32–2.81)	584	2.74	(2.48–3.03)	0.31
Skin, squamous cell	8369	426	2.07	(1.86–2.29)	189	2.16	(1.85–2.52)	104	2.25	(1.83–2.77)	0.75
Nervous system	19099	371	1.54	(1.35–1.75)	349	1.58	(1.38–1.80)	215	1.71	(1.44–2.03)	0.45
Thyroid gland	4791	90	5.13	(3.91–6.74)	87	5.61	(4.25–7.40)	48	5.43	(3.74–7.87)	0.86
Endocrine glands	7810	130	2.05	(1.61–2.62)	110	1.99	(1.53–2.59)	60	1.98	(1.38–2.83)	0.98
Non–Hodgkin lymphoma	11895	276	1.61	(1.41–1.84)	203	1.65	(1.41–1.93)	124	1.69	(1.38–2.06)	0.85
Hodgkin lymphoma	3931	19	2.57	(1.65–3.98)	18	2.59	(1.65–4.07)	40	9.60	(7.08–13.00)	**<.0001**
Myeloma	3296	61	2.12	(1.64–2.74)	40	1.96	(1.43–2.68)	22	2.73	(1.79–4.18)	0.21
Leukemia	12149	277	1.86	(1.62–2.14)	216	1.88	(1.61–2.20)	105	2.17	(1.73–2.71)	0.31

^1^Individuals were only considered to have a positive family history if the parental cancer was diagnosed at age ≤ 78 years.

^2^Risk estimates for limited parental age were considered for comparison.

^3^N = Number of cases with a concordant cancer in the family.

^4^RR = Relative risk.

^5^CI = Confidence interval.

**Table 2:** Familial risk for cancers diagnosed at early and late ages.

Cancer site	FDRs^1^ diagnosed at age < 60 years						FDRs diagnosed at age ≥ 60 years						p-value for differences in RRs among all groups^2^
	Index individuals at age < 60 years			Index individuals at age ≥ 60 years			Index individuals at age < 60 years			Index individuals at age ≥ 60 years			
	N^3^	RR^4^	95% CI^5^	N	RR	95% CI	N	RR	95% CI	N	RR	95% CI	
Upper aerodigestive tract	38	1.65	(0.92–2.99)	32	2.74	(1.44–5.22)	79	1.55	(1.03–2.34)	51	1.36	(0.81–2.27)	0.44
Esophagus	4	3.70	(1.02–13.50)	6	5.55	(1.93–16.00)	10	1.98	(0.87–4.49)	17	2.45	(1.30–4.59)	0.51
Stomach	39	2.70	(1.91–3.82)	33	3.14	(2.16–4.58)	94	1.72	(1.37–2.15)	107	1.69	(1.36–2.09)	**0.0084**
Small intestine	6	7.89	(3.54–17.60)	5	12.08	(5.02–29.10)	12	5.81	(3.29–10.30)	10	5.97	(3.20–11.10)	0.57
Colorectum	529	3.14	(2.82–3.49)	286	2.00	(1.74–2.31)	1563	1.80	(1.69–1.92)	1780	1.64	(1.54–1.74)	**<.0001**
Liver	16	2.19	(1.20–4.00)	10	1.75	(0.82–3.73)	56	1.55	(1.12–2.14)	78	1.67	(1.27–2.21)	0.81
Pancreas	39	4.47	(3.14–6.37)	18	2.14	(1.28–3.60)	81	2.15	(1.68–2.76)	102	1.82	(1.46–2.28)	**0.0016**
Lung	274	2.77	(2.39–3.22)	229	2.31	(1.96–2.71)	707	1.95	(1.78–2.15)	937	1.96	(1.81–2.13)	**0.0003**
Breast	3854	2.15	(2.05–2.26)	1118	1.79	(1.64–1.96)	3730	1.67	(1.59–1.75)	1659	1.59	(1.48–1.71)	**<.0001**
Cervix	134	1.85	(1.50–2.27)	4	0.72	(0.22–2.37)	39	1.48	(1.01–2.16)	3	0.88	(0.22–3.48)	0.18
Endometrium	149	4.31	(3.43–5.42)	56	1.89	(1.31–2.73)	120	1.70	(1.32–2.19)	127	1.97	(1.54–2.52)	**<.0001**
Ovary	197	4.23	(3.65–4.90)	39	2.53	(1.82–3.52)	116	2.03	(1.68–2.45)	43	1.64	(1.20–2.24)	**<.0001**
Prostate	673	7.21	(6.56–7.93)	916	3.51	(3.24–3.80)	2727	2.79	(2.64–2.94)	7300	2.13	(2.06–2.19)	**<.0001**
Testis	118	6.25	(5.12–7.63)	1			4			0			
Kidney	80	3.05	(2.33–3.98)	36	2.22	(1.49–3.31)	98	1.53	(1.20–1.95)	74	1.29	(0.98–1.71)	**<.0001**
Urinary bladder	75	2.19	(1.63–2.94)	60	1.83	(1.32–2.55)	250	1.67	(1.42–1.97)	327	1.90	(1.64–2.20)	0.41
Melanoma	652	3.13	(2.84–3.44)	117	2.64	(2.11–3.31)	477	2.19	(1.96–2.45)	185	2.71	(2.26–3.25)	**<.0001**
Skin, squamous cell	29	2.20	(1.47–3.31)	25	1.88	(1.21–2.90)	201	2.02	(1.73–2.37)	294	2.24	(1.97–2.56)	0.7
Nervous system	326	1.97	(1.68–2.32)	42	1.47	(0.94–2.30)	167	1.27	(1.01–1.59)	78	1.79	(1.29–2.49)	**0.0121**
Thyroid gland	132	9.20	(7.43–11.40)	8	5.77	(2.45–13.60)	33	3.57	(2.34–5.45)	3	1.77	(0.44–7.14)	**<.0001**
Endocrine glands	114	3.53	(2.77–4.51)	11	1.41	(0.65–3.09)	63	1.55	(1.12–2.16)	28	2.00	(1.22–3.27)	**0.0003**
Non–Hodgkin lymphoma	82	1.76	(1.37–2.26)	39	1.84	(1.28–2.64)	174	1.61	(1.35–1.91)	112	1.57	(1.27–1.95)	0.83
Hodgkin lymphoma	57	7.26	(5.66–9.31)	2			4			0			
Myeloma	4	1.21	(0.47–3.10)	6	2.13	(0.99–4.59)	31	2.23	(1.59–3.14)	42	2.48	(1.85–3.33)	0.47
Leukemia	92	2.09	(1.62–2.69)	32	2.21	(1.44–3.39)	145	1.80	(1.47–2.20)	133	2.25	(1.82–2.79)	0.48

^1^FDR = First-degree relative.

^2^P-value based on likelihood ratio test.

^3^N = Number of concordant cases.

^4^RR = Relative risk.

^5^CI = Confidence interval.

**Table 3:** Familial cancer risks for people with multiple affected relatives.

	Family history	Cancer site						
		Colorectum	Lung	Breast	Prostate	Urinarybladder	Melanoma	Nervous system
**Case numbers**^1^**, Relative risk, 95%CI**	Both parents affected	63	25	11	0	6	8	3
		1.98 (1.46–2.69)	3.02 (1.86–4.92)	9.49 (4.58–19.67)		3.50 (0.92–13.28)	6.00 (2.54–14.15)	3.64 (0.82–16.17)
	1 parent and 1 sibling affected	186	42	485	930	12	50	24
		3.67 (3.07–4.39)	2.48 (1.70–3.61)	2.88 (2.58–3.21)	4.59 (4.23–4.98)	2.86 (1.11–7.35)	8.20 (5.81–11.56)	9.47 (5.59–16.05)
	2 siblings affected	33	27	199	368	3	15	0
		2.57 (1.68–3.91)	3.39 (2.12–5.42)	2.50 (2.11–2.97)	5.25 (4.62–5.98)	2.74 (0.42–18.03)	4.47 (2.39–8.37)	
	3 siblings affected	4	4	4	44	0	0	0
		6.59 (1.97–22.08)	20.84 (6.17–70.38)	1.02 (0.30–3.41)	6.78 (4.68–9.83)			
**P-values for differences among RRs**^2^	Both parents vs. 1 parent	0.36	0.0528	**<.0001**		0.31	**0.0398**	0.26
	1 Parent and 1 sibling vs. 1 parent	**<.0001**	0.14	**<.0001**	**<.0001**	0.32	**<.0001**	**<.0001**
	1 Parent and 1 sibling vs. 1 sibling	**<.0001**	0.95	**<.0001**	**<.0001**	0.41	**<.0001**	**<.0001**
	2 siblings vs. 1 sibling	0.25	0.22	**0.0006**	**<.0001**	0.71	0.13	
	3 siblings vs. 1 sibling	0.0528	**0.0007**	0.34	**<.0001**			
	3 siblings vs. 2 sibling	0.15	**0.0063**	0.15	0.20			
	1 Parent and 1 sibling vs. 2 siblings	0.12	0.31	0.18	0.08	0.97	0.10	
	1 Parent and 1 sibling vs. both parents	**0.0006**	0.53	**0.0015**		0.81	0.51	0.24
	2 siblings vs. both parents	0.33	0.74	**0.0005**		0.83	0.59	

^1^Number of concordant cases

^2^P-values from Wald chi-square statistics testing for pairwise differences among RRs for different family histories.

**Table 4:** Number and percentage of cases with and without a concordant cancer in the family among individuals at age ≥ 60 years.

Cancer site	Number of cases (%) with a negative family history	Number of cases (%) with concordant cancers among first-degree relatives	
		2 affected FDR^1^	3 or more affected FDRs
Upper aerodigestive tract	2733 (97.1)	81 (2.9)	2 (0.1)
Esophagus	1251 (98.2)	23 (1.8)	0 (0)
Stomach	1958 (93.3)	133 (6.3)	7 (0.3)
Small intestine	611 (97.6)	14 (2.2)	1 (0.2)
Colorectum	12934 (86.2)	1940 (12.9)	126 (0.8)
Liver	2280 (96.3)	87 (3.7)	1 (0.0)
Pancreas	2823 (95.9)	116(3.9)	4 (0.1)
Lung	10215 (89.8)	1124 (9.9)	42 (0.4)
Breast	16469 (85.6)	2573 (13.4)	204 (1.1)
Cervix	623 (98.9)	7 (1.1)	0 (0)
Endometrium	4458 (96.1)	179 (3.9)	4 (0.1)
Ovary	2336 (96.6)	80 (3.3)	2 (0.1)
Prostate	29273 (78.1)	7148 (19.1)	1071 (2.9)
Testis	108 (99.1)	1 (0.9)	0 (0)
Kidney	3099 (96.6)	108 (3.4)	2 (0.1)
Urinary bladder	5711 (93.7)	378 (6.2)	9 (0.2)
Melanoma	5251 (94.5)	288 (5.2)	15 (0.3)
Skin, squamous cell	4564 (93.5)	307 (6.3)	12 (0.3)
Nervous system	3364 (96.6)	117 (3.4)	3 (0.1)
Thyroid gland	504 (97.9)	11 (2.1)	0 (0)
Endocrine glands	1764 (97.8)	37 (2.1)	2 (0.1)
Non-Hodgkin lymphoma	4072(96.4)	148 (3.5)	3 (0.1)
Hodgkin lymphoma	222 (99.1)	2 (0.9)	0 (0)
Myeloma	1690 (97.2)	48 (2.8)	0 (0)
Leukemia	3366 (95.3)	162 (4.6)	3 (0.1)

^1^FDR = First-degree relative.
